# Atypical pantothenate kinase-associated neurodegeneration with variable phenotypes in an Egyptian family

**DOI:** 10.1016/j.heliyon.2021.e07469

**Published:** 2021-07-02

**Authors:** Ali S. Shalash, Thomas W. Rösler, Ibrahim Y. Abdelrahman, Hatem S. Abulmakarem, Stefanie H. Müller, Franziska Hopfner, Gregor Kuhlenbäumer, Günter U. Höglinger, Mohamed Salama

**Affiliations:** a Department of Neurology, Faculty of Medicine, Ain Shams University, Cairo, Egypt; b Department of Neurology, School of Medicine, Technical University Munich, Munich, Germany; c Department of Translational Neurodegeneration, German Center for Neurodegenerative Diseases (DZNE), Munich, Germany; d Radiation Biology Department, National Center for Radiation Research and Technology, Egyptian Atomic Energy Authority, Cairo, Egypt; e Institute of Health Informatics, UCL, London, UK; f Department of Neurology, Hannover Medical School, Hannover, Germany; g Department of Neurology, Kiel University, Kiel, Germany; h Institute of Global Health and Human Ecology, American University in Cairo (AUC), Cairo, Egypt; i Faculty of Medicine, Mansoura University, Mansoura, Egypt

**Keywords:** Pantothenate kinase-associated neurodegeneration, *PANK2*, Neurodegeneration iron accumulation, Genetics, Dystonia, Parkinsonism

## Abstract

Pantothenate kinase-associated neurodegeneration (PKAN) is a rare hereditary neurodegenerative disease characterized by an accumulation of iron within the brain. In the present report, we describe a family with 4 affected siblings presenting with variable clinical manifestations, e.g., parkinsonian features, dystonia and slow disease progression over 5 years. Exome sequencing revealed a causative variant in the pantothenate kinase 2 gene (*PANK2*). Variant NM_024960.6:c.710C > T was homozygous in all affected subjects. Our report describes the first genetically confirmed cases of PKAN in the Egyptian population. Studying genetics of neurodegenerative diseases in different ethnicities is very important for determining clinical phenotypes and understanding pathomechanisms of these diseases.

## 1 Introduction

Pantothenate kinase-associated neurodegeneration (PKAN) is a rare neurodegenerative disease characterized by abnormal accumulation of iron in distinct brain areas. It has an estimated worldwide incidence of 2 affected individuals in 1 million which is even lower among the African population (Brezavar and Bonnen 2019). However, in the heterogenous group of neurodegenerative disorders with brain iron accumulation (NBIA), it is considered to be the most common (Hayflick et al., 2003; Hartig et al., 2006). PKAN is a genetically inheritable disease following an autosomal recessive pattern with causative mutations in the pantothenate kinase 2 (*PANK2*) gene. Pantothenate kinases control the biosynthesis of the coenzyme A (Zhou et al., 2001).

Clinically, PKAN shows a wide range of signs and symptoms including motor manifestations, e.g., dystonia, parkinsonism, dysarthria, dysphagia and spasticity, psychiatric and cognitive impairment as well as oculomotor disturbances (Lee et al., 2016). According to age of onset and disease progression, PKAN is classified into typical and atypical types. Atypical PKAN is characterized by later onset and slower progression (Hartig et al., 2006). Genetic determinants of PKAN, however, are not well characterized. Additionally, the current knowledge about PKAN is mainly built on individual case reports and case series (Marshall et al., 2019). The disease progression, severity of symptoms and even correlation between specific mutations and the disease phenotype is highly variable (Tomic et al., 2015).

Neurogenetic studies about PKAN are scarce in North Africa. So far, only one case report described a female patient with PKAN in a consanguineous Moroccan family, identifying a causative homozygous deletion in *PANK2* (Efthymiou et al., 2020). In the present study, we describe an Egyptian family with 4 affected members revealing slow progression of atypical PKAN. The patients had different ages of onset and showed variable clinical presentations (parkinsonism, dystonia), normal serum ferritin levels, absence of acanthocytes, the brain MRI characteristic eye-of-the-tiger sign, all caused by the same homozygous mutation in *PANK2*.

## 2 Materials and methods

### 2.1 Ethics statement

The family presented to the Movement Disorders Clinic, Department of Neurology at Ain Shams University, Cairo, where six members (affected and healthy) were examined by three movement disorders experts (A.S.S., F.H. and G.U.H.). Blood samples were collected from healthy and affected members. Standard brain MRI at 1.5 T with T1-, T2-, and FLAIR-sequences of patients II.3 and II.10 was performed. Ethical approval was obtained from Mansoura University, Egypt (RP/42) and Technical University of Munich, Germany (203/15s). All participants provided an informed consent.

### 2.2 Cases

**Figure 1:**
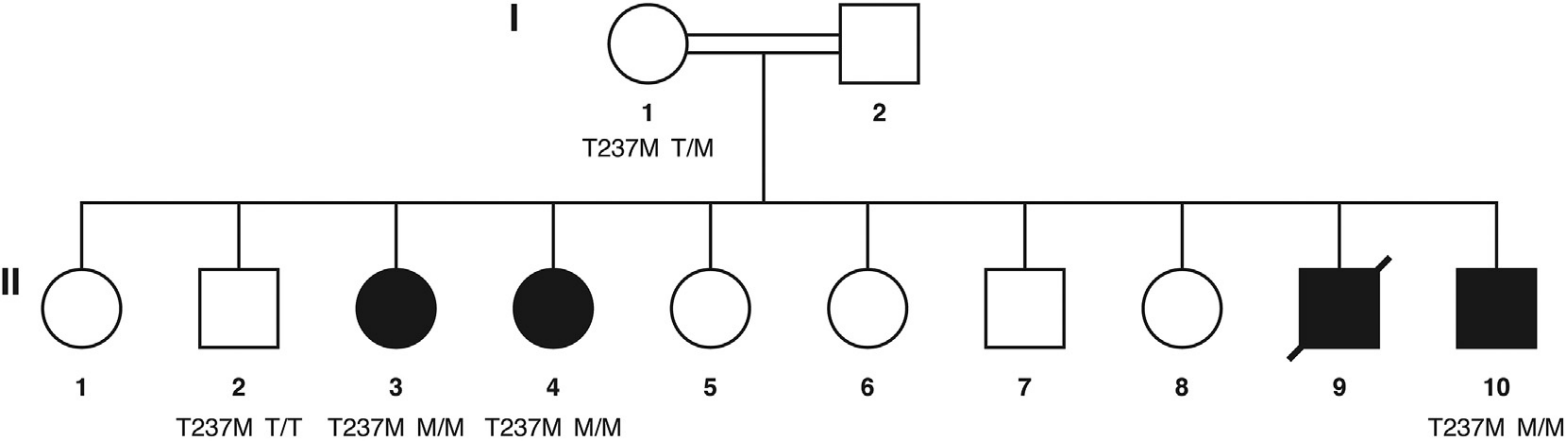
Pedigree of the examined family. The unaffected consanguineous parents (double line) had 10 children. Four children developed PKAN (black filling), two female (circle) and two male (square), of whom one already had deceased (diagonal slash). The genotypes of *PANK2* variant NM_024960.6:c.710C > T are shown for all family members who provided DNA.

The examined family consisted of fourth-degree consanguineous parents and 10 children (see Figure 1). The father had a history of psychiatric illness (psychosis). In total, four children were affected. Patient II.3 presented at age 38 mainly parkinsonian features (i.e., bradykinesia, rigidity and kinetic tremor), which had started at age 30, and were followed by jaw-opening dystonia and depression. Patients II.4 and II.10 were presented at ages 32 and 17 respectively with generalized dystonia including jaw-opening dystonia, upper limb dystonia, associated with dysarthria, agitation, and behavioral changes. Age at symptom onset of patients II.4 and II.10 was 15 and 7 years, respectively. Patient II.9 died at age 23. He had a history of forced eyelid closure and jaw-opening dystonia which started at age 18. The patients were re-assessed 5 years after the initial clinical examination and showed mild progression of their symptoms with normal or minimally impaired gait (see Table 1).

**Table 1:** Clinical characteristics of PKAN patients.

Features	Patient II.3	Patient II.4	Patient II.10
Sex	Female	Female	Male
Age at presentation (y)	38	32	17
Age of onset (y)	30	15	7
Disease duration (y)	8	17	10
Symptoms at onset	Dysarthria, UL bradykinesia	Tongue protrusion, jaw-opening dystonia	Behavioral changes, jaw-opening dystonia
Jaw-opening dystonia	Mild, sensory tricks	Present	Severe
Tongue protrusion	-	Repeated	Occasional
Limb dystonia	-	Present; more in UL (generalized dystonia)	Present; more in UL (generalized dystonia)
Dysarthria	Present	Present	Severe (anarthria)
Dysphagia	-	Present	Present
Stuttering	Present	-	-
Choking	-	Present	Present
Sialorrhea	-	-	Present
Parkinsonism	Present; symmetrical bradykinesia, rigidity, kinetic tremor, mainly UL, no response to levodopa	Present; mild rigidity of UL	-
Pyramidal weakness	Present	Present	-
Spasticity	Present	Present	-
Plantar reflex	Extensor	Extensor	Flexor
Deep tendon reflexes	Exaggerated	Exaggerated	Exaggerated
Depression	Present	Present	-
Anxiety	Present	Present	-
Agitation	Occasional	Occasional	Present
Behavioral changes	-	-	Present
Cognition	Average (MMSE 26/30)	Impaired (MMSE 14/27)	No reported impairment
Fundus	Normal	Normal	Normal
Serum ferritin	Normal (188 ng/mL)	Normal (210 ng/mL)	Normal (199.5 ng/mL)
Blood acanthocytosis	Absent	Absent	Absent
Brain MRI scan	Eye-of-the-tiger-sign	Not available	Eye-of-the-tiger-sign
Course of disease	Slowly progressing, mild worsening of Jaw-opening dystonia	Slowly progressing	Slowly progressing, mild worsening of LL dystonia

UL, upper limbs; LL, lower limbs.

**Figure 2:**
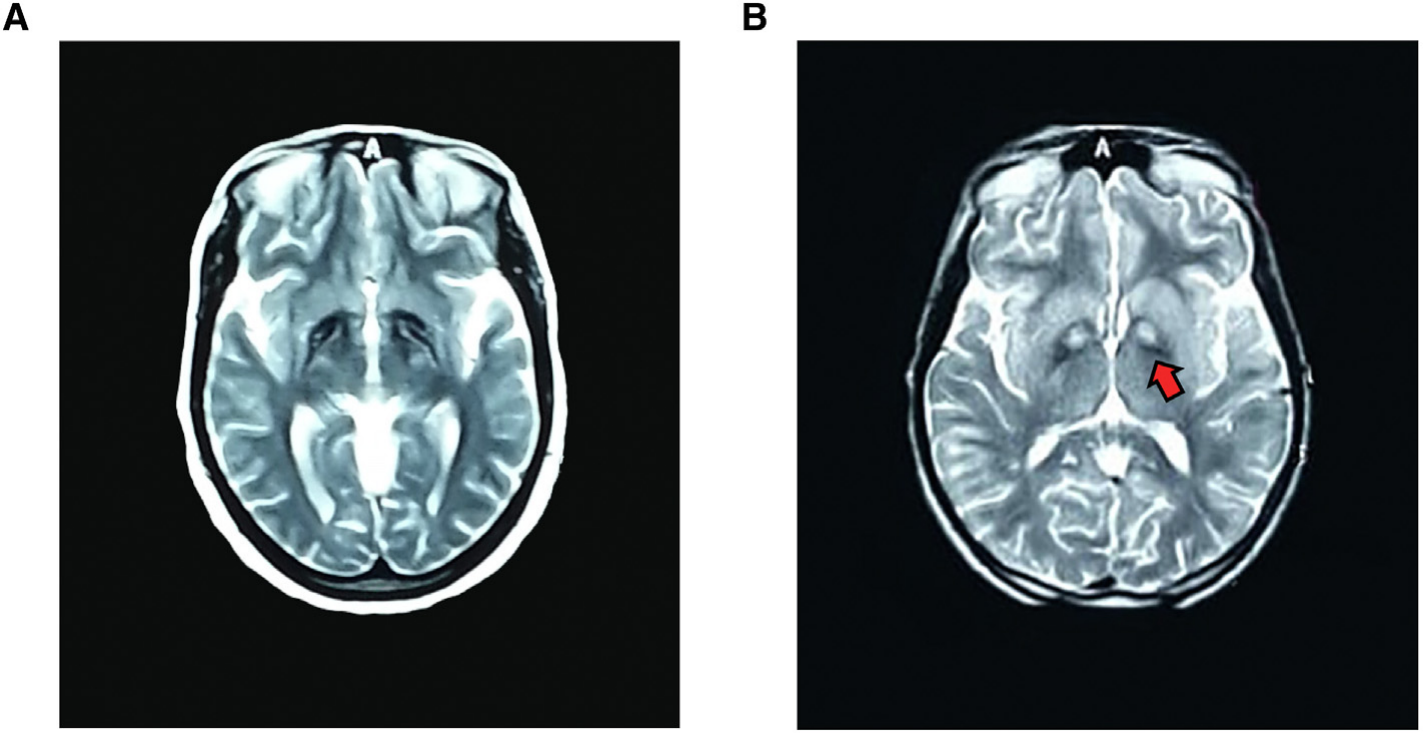
Brain MRI scans of PKAN-affected family members. Images show axial brain MRI scans of patient II.3 (A, FLAIR) and II.10 (B, T2WI) with hyperintensity surrounded by hypointensity of globus pallidus with the characteristic eye-of-the-tiger sign (red arrow).

All patients had normal serum ferritin levels and did not show blood acanthocytosis. Brain magnetic resonance imaging (MRI) was done for patients II.3 and II.10 revealing the characteristic eye-of-the-tiger sign (see Figure 2a, b).

### 2.3 Genetic analyses

Exome sequencing was performed in the two affected individuals II:4 and II:10. Genomic DNA libraries were captured using the Nextera Rapid Capture Expanded Exome Kit (Illumina, San Diego, CA), and DNA fragments were sequenced on an Illumina HiSeq2000 system with an average coverage of 80x. Variants were identified by a standard analysis pipeline and annotated using ANNOVAR (Wang et al., 2010). We discarded variants with a minor allele frequency (MAF) > 0.01 in gnomAD (Genome Aggregation Database, https://gnomad.broadinstitute.org) “all” exome as well as genome data. We discarded variants without an annotated exonic or splicing function and variants with a CADD (Combined Annotation Dependent Depletion) score below 15 (Kircher et al., 2014). Since the parents are related, we assumed autosomal recessive inheritance and homozygosity of the causative variant. However, to guarantee of not missing any variants of importance, we filtered for variants present in both II:4 and II:10 but not for homozygosity. We filtered for specific genes with the symbols *ATP13A2*, *C19orf12*, *COASY*, *CP*, *DCAF17*, *FAH2*, *FTL*, *PANK2*, *PLA2G6*, *WDR45* known to harbor variants causing NBIA and related phenotypes. The filtering resulted in only one homozygous variant in the *PANK2* gene, NM_024960.6:c.710C > T [ a pathogenic variant ] causing the amino acid change NP_079236.3:p.Thr237Met in the PANK2 protein in both exome sequenced affected family members (see Table 2 and Figure 1). Segregation of this variant was ascertained by Sanger sequencing of all family members of whom DNA was available. We confirmed the presence of the variant and showed that patient II:3 also carries this variant in the homozygous state. The mother I:1 is a heterozygous carrier of the variant and her healthy son II:2 did not show this variant (see Figure 1). DNA samples of the father I:2, the deceased affected brother II:9 and other siblings were not available for analysis.

**Table 2:** Identified variant in the PANK2 gene (GRCh38/hg38).

Genetic finding	Variant
Chromosome level	chr20.hg38:g.3918717C > T
Genomic level	NC_000020.11:g.3918717C > T
Coding sequence level	NM_024960.6:c.710C > T
Protein level	NP_079236.3:p.Thr237Met
CADD (Phred-scaled)	22
MutationTaster (score/class)	0.926/deleterious
PolyPhen-2 HVAR (score/class)	0.242/benign
gnomAD exomes (MAF, nr. of alleles analyzed)	0.000012
dbSNP (153 all)	rs137852967
HGMD (public 01.08.21)	Not listed
ClinVar	Pathogenic, multiple submitters, no conflict

CADD, Combined Annotation Dependent Depletion; dbSNP, database of single nucleotide polymorphism; gnomAD, Genome Aggregation Database; HGMD, Human Gene Mutation Database; MAF, minor allele frequency.

The effects of amino acid substitutions on protein function were predicted using MutationTaster (Schwarz et al., 2014), PolyPhen-2 (Adzhubei et al., 2010), and CADD (Rentzsch et al., 2019). Furthermore, we searched the public version of the Human Gene Mutation Database (Stenson et al., 2020) and ClinVar (Landrum et al., 2018) for the variant (see Table 2).

## 3 Discussion

In the last two decades, genetic research in neurodegenerative diseases has tremendously progressed, especially through focused analyses of families with Mendelian mode of inheritance in different populations. However, such progress is not globally oriented. Including more genetic findings from understudied populations such as Africans, will help to identify variable phenotypes, enhance discoveries and offer better understanding of the diseases’ pathophysiology and genotype-phenotype correlation.

Our report confirms the pathogenicity of the *PANK2* variant NM_024960.6:c.710C > T which causes PKAN. Although this variant has been described before, this is the first report of a pathogenic *PANK2* variant in the Egyptian population. Homozygous and compound heterozygous variants in *PANK2* have been identified as the most common cause of NBIA (Zhou et al., 2001; Hayflick et al., 2003). The identified variant has been reported by multiple submitters to the ClinVar database (Landrum et al., 2018) as pathogenic and can be found in other published genetic studies of NBIA (Hartig et al., 2006) but is not listed in the public version of the HGMD (Human Gene Mutation Database). Because the homozygous state of this variant segregates in the examined Egyptian family with the disease and has already been shown to be pathogenic, it could be the cause of NBIA in this Egyptian family.

Several variants in PANK2 have been identified in PKAN cases. The most common is the *PANK2* variant p.Gly521Arg which accounts for approximately 30% of cases (Zhou et al., 2001). This variant leads to a catalytically inactive PANK2 protein due to improper folding (Zhang et al., 2006). As PANK2 is localized to the mitochondria where it is a key enzyme in the biosynthesis of coenzyme A (Leonardi et al., 2007), influencing important metabolic processes, nonfunctional PANK2 proteins have a tremendous influence on cell energy processes. However, the PANK2 variant p.Thr237Met which we have identified in the Egyptian family is less common for PKAN and does not induce catalytic or regulatory deficits (Zhang et al., 2006). This implies that other presently unknown effects and functions of this variant might contribute to its pathogenicity.

Remarkably, the identified *PANK2* mutation led to variable phenotypes and ages of onset within the same family. Previous studies reported the mutation to cause predominant jaw-opening dystonia, limb dystonia, and dysarthria (Hartig et al., 2006; Tomic et al., 2015; Yapici et al., 2016). Predominant parkinsonism was less commonly reported (Chang et al., 2020). However, in the present family we could observe combinations of these symptoms. The affected members showed early cranial symptoms and a slow course of disease progression compared to previous reports (Tomic et al., 2015). This milder course of late onset PKAN revealed no contractures and preserved ambulation, however, further follow-up is required. Our finding of a clinical variability within the reported family confirms the relevance of atypical PKAN as a differential diagnosis for familial movement disorders with variable phenotypes such as Wilson's disease (Schneider et al., 2006; Shalash et al., 2014).

## 4 Conclusion

The current report describes variable clinical phenotypes and disease progression of atypical PKAN in affected members of an Egyptian family. To the best of our knowledge, it is the second report of a family with PKAN in Africa. These findings add to our knowledge about the genetics of this disease in North Africa.

## Declarations

### Author contribution statement

Ali S. Shalash, Thomas W. Rösler, Gregor Kuhlenbäumer, Mohamed Salama: Conceived and designed the experiments; Performed the experiments; Analyzed and interpreted the data; Wrote the paper.

Stefanie H. Müller, Franziska Hopfner, Günter U. Höglinger: Conceived and designed the experiments; Performed the experiments; Analyzed and interpreted the data.

Ibrahim Y. Abdelrahman, Hatem S. Abulmakarem: Conceived and designed the experiments.

### Funding statement

This work was supported by the 10.13039/501100001655German Academic Exchange Service (DAAD) through the funding programme “Higher Education Dialogue with the Muslim World”, project “GeneFINDER”.

This work was also supported by the Institute of Global Health and Human Ecology (I-GHHE) at the 10.13039/501100009229American University in Cairo (AUC), Egypt.

### Data availability statement

Data will be made available on request.

### Declaration of interests statement

The authors declare no conflict of interest.

### Additional information

No additional information is available for this paper.
